# Genome-wide identification and expression analysis of the HVA22 gene family in cotton and functional analysis of *GhHVA22E1D* in drought and salt tolerance

**DOI:** 10.3389/fpls.2023.1139526

**Published:** 2023-03-06

**Authors:** Haijun Zhang, Yanchao Yuan, Huixian Xing, Ming Xin, Muhammad Saeed, Qi Wu, Jing Wu, Tao Zhuang, Xiaopei Zhang, Lili Mao, Xuezhen Sun, Xianliang Song, Zongwen Wang

**Affiliations:** ^1^ State Key Laboratory of Crop Biology/Agronomy College, Shandong Agricultural University, Taian, China; ^2^ College of Life Sciences, Qingdao Agricultural University, Key Lab of Plant Biotechnology in Universities of Shandong Province, Qingdao, China; ^3^ College of Tropical Crops, Hainan University, Haikou, China; ^4^ Department of Agricultural Sciences, College of Agriculture and Environmental Sciences, Faculty of Life Sciences, Government College University Faisalabad, Faisalabad, Pakistan; ^5^ Institute of Industrial Crops, Shandong Academy of Agricultural Sciences, Jinan, China

**Keywords:** cotton, *HVA22s*, salt, overexpression, VIGS, drought, stress

## Abstract

The HVA22 family of genes, induced by abscisic acid and stress, encodes a class of stress response proteins with a conserved TB2/DP1/HVA22 domain that are unique among eukaryotes. Previous studies have shown that *HVA22s* play an important role in plant responses to abiotic stresses. In the present study, 34, 32, 16, and 17 *HVA22s* were identified in *G. barbadense*, *G. hirsutum*, *G. arboreum*, and *G. raimondii*, respectively. These *HVA22* genes were classified into nine subgroups, randomly distributed on the chromosomes. Synteny analysis showed that the amplification of the *HVA22s* were mainly due to segmental duplication or whole genome replication (WGD). Most *HVA22s* promoter sequences contain a large number of drought response elements (MYB), defense and stress response elements (TC-rich repeats), and hormone response elements (ABRE, ERE, SARE, etc.), suggesting that *HVA22s* may respond to adversity stresses. Expression profiling demonstrated that most *GhHVA22s* showed a constitutive expression pattern in *G. hirsutum* and could respond to abiotic stresses such as salt, drought, and low temperature. Overexpression of *GhHVA22E1D* (*GH_D07G0564*) in *Arabidopsis thaliana* enhances salt and drought tolerance in Arabidopsis. Virus-induced gene silencing of *GhHVA22E1D* reduced salt and drought tolerance in cotton. This indicates that *GhHVA22E1D* plays an active role in the plant response to salt stress and drought stress. *GhHVA22E1D* may act in plant response to adversity by altering the antioxidant capacity of plants. This study provides valuable information for the functional genomic study of the HVA22 gene family in cotton. It also provides a reference for further elucidation of the functional studies of HVA22 in plant resistance to abiotic stress response.

## Introduction

Plants are easily affected by environmental stresses during growth due to their immobility, and salt stress and drought stress are the two major abiotic stress factors that seriously affect plant growth (Magwanga et al., 2019a; Magwanga et al., 2019b). Plants have evolved a range of regulatory mechanisms to cope in response to environmental stresses, for example, osmotic regulation, antioxidant defense regulation, signal transduction mechanisms, etc. The abscisic acid (ABA) signaling pathway plays an important part in the response of plants to abiotic stresses (Campos-Rivero et al., 2017; Liu et al., 2020). ABA regulates plant physiological and metabolic responses by controlling the expression levels of many stress-responsive genes, enabling plants to tolerate stresses (Ma et al., 2018; Zhang et al., 2021). *HVA22* is a unique ABA-induced gene, which was first discovered in *Hordeum vulgare* L. aleurone cells (Shen et al., 1993). There was a high sequence similarity between plant HVA22s and human TB2/DP1 proteins. HVA22 family proteins with conserved TB2/DP1/HVA22 domains were ubiquitous in eukaryotes, but were not found in prokaryotes(Sharon, 2017). *HvHVA22* helps to regulate vesicular transport in stressed cells and reduces the non-essential secretion of stressed cells, which results in improved plant resistance (Guo and David Ho, 2008).

In previous studies, multiple functions of *HVA22s* have been identified. *HvHVA22* negatively regulates GA (Gibberellic acid)-mediated programmed cell death and vacuolation in barley dextrin cells (Guo and David Ho, 2008). Deletion of *AtHVA22d* leads to enhanced autophagy and impaired flower development in *Arabidopsis thaliana* (Chen et al., 2009). Overexpression of *OsHLP1* (HVA22-like protein 1) significantly enhances blast disease resistance by impairing endoplasmic reticulum homeostasis in *Oryza sativa* (Meng et al., 2022). Furthermore, numerous works have shown that *HVA22s* has abiotic stress response function. *HvHVA22s* and *AtHVA22s* were up-regulated in in barley and *A. thaliana*, respectively, to cope with various environmental stresses, such as salinity, drought, cold, and exogenous ABA (Shen et al., 1993; Shen et al., 2001; Chen et al., 2002; Collin et al., 2020). The *ZmHVA22* in *Zea mays* was down-regulated significantly under high salt, simulated drought and cold stress, while the *ZmHVA22* was up-regulated to varying degrees under high temperature stress, ethylene induction, and ABA(Chen et al., 2014). The expression of *SaHVA22* in *Spartina alterniflora* was significantly higher when treated with a salt concentration of 68 mM than other salt concentration treatments (0 mM, 137 mM, 205 mM) (Courtney et al., 2016). Moreover, the expression of *HVA22s* was up-regulated in *Vicia faba* under cold stress (Lyu et al., 2021), *G. hirsutum* under salinity stress (Yuan et al., 2019) and *Solanum tuberosum* under drought stress (Benny et al., 2019).

In previous studies, the potential of *HVA22s* in abiotic stress had been emphasized, and systematic family analysis of *HVA22s* could help to comprehend their functional properties better. Up to now, the *HVA22* family has been systematically analyzed merely in *Arabidopsis thaliana*, *Solanum lycopersicum*, *Citrus clementina* and *Citrus sinensis*, and 5, 15, 6 and 6 *HVA22s* had been identified, respectively (Chen et al., 2002; Gomes Ferreira et al., 2019; Wai et al., 2022). The five *AtHVA22s* in *A. thaliana* could be divided into two subfamilies; thereinto, the sequences of *AtHVA22d* and *AtHVA22e* were closer to *HVA22* in barley (Chen et al., 2002). The *HVA22s* of tomato and citrus were divided into four subfamilies according to the sequence similarity with *AtHVA22s* (Gomes Ferreira et al., 2019; Wai et al., 2022).

Cotton is a vital source of natural fiber crops in the world and one of the main sources of plant protein and plant oil, and it is one of the important strategic materials in China (Liu et al., 2022). Around 1.6 million years ago, the A_0_-genome of an extinct diploid cotton and the D_5_-genome of the diploid cotton *Gossypium raimondiid* were crossed and doubled to form a heterotetraploid species (Grover et al., 2015; Huang et al., 2020; Peng et al., 2022). This tetraploid specie then diverged subsequently into seven tetraploid cottons namely *G. hirsutum*, *G. barbadense*, *G. tomentosum*, *G. mustelinum*, *G. darwinii*, *G. ekmanianum*, and *G. stephensii*, and the genomes of these seven tetraploid cotton species were labeled as (AD)_1_ to (AD)_7_ (Chen et al., 2020b; Peng et al., 2022). Among them, *G. hirsutum* and *G. barbadense* were domesticated as cultivated species, the two cultivated allotetraploid cotton species with quite different traits in morphology, yield, fiber quality, environmental adaptability, and genomic sequences (Hu et al., 2019). *G. herbaceum* (A_1_) and *G. arboreum* (A_2_) were formed about 0.7 million years ago by A_0_-genome divergence (Huang et al., 2020). At present, the genomes of *G. barbadense*, *G. hirsutum*, *G. arboreum*, and *G. raimondii* had been well sequenced (Paterson et al., 2012; Du et al., 2018; Hu et al., 2019; Huang et al., 2020); this greatly facilitates the determination of *HVA22* gene family members in cotton and clarification of their evolutionary relationships.

In this study, the *HVA22s* was identified from *G. raimondii*, *G. arboreum*, *G. hirsutum*, and *G. barbadense*. The characteristics of gene structure, subcellular localization, conserved motifs and domains, evolution, synteny relationship, chromosome localization, and expression patterns were systematically characterized and analyzed. Furthermore, the distribution of cis-acting elements in the promoters of the *HVA22* family was also analyzed, which has an important role in our further understanding of the function of *HVA22s*. Previous studies had identified the *GhHVA22E1D* gene as a differentially expressed gene under salt stress through a whole-genome association and differential expression analysis of salt tolerance in *G. hirsutum* during germination (Yuan et al., 2019). This study has shown that overexpression of *GhHVA22E1D*, the *HVA22* family member of *G. hirsutum*, improved the drought resistance and salt tolerance in *A. thaliana*. Meanwhile, silencing of *GhHVA22E1D* reduced salt tolerance and drought resistance in cotton. In general, the results of this research provided a reference for further studies to understand the action of *HVA22s* in cotton.

## Materials and methods

### Database and sequence retrieval

The genome and annotation files of *G. barbadense* (H7124, ZJU), *G. hirsutum* (TM-1, ZJU), *G. raimondii* (JGI), and *G. arboreum* (Shiyaxi1 CRI) were downloaded from CottonFGD (https://cottonfgd.net/). *A. thaliana* HVA22 protein sequences (Chen et al., 2002) were downloaded from TAIR online website (http://www.arabidopsis.org/index.jsp).

### Identification and genetic characterization of *HVA22* family members

All the five *A. thaliana* HVA22 protein sequences were taken as reference sequences for comparison. BLASTP search was used to scan the whole-genome protein sequences of the four cotton species. The Hidden Markov Model (HMM) profile of the HVA22 domain (PF03134) was obtained from the Pfam website (http://pfam.xfam.org/). The HMM search was used to identify HVA22. The NCBI Conserved Domain Database (CDD: https://www.ncbi.nlm.nih.gov/cdd), using an automated model and default parameters (maximum number of hits = 500, threshold = 0.01), was used to subject the putative HVA22 protein sequences to HVA22 conserved structural domain validation, removing the putative HVA22 protein sequence that did not contain the TB2/DP1/HVA22 domain.

The basic information and physical and chemical properties of all HVA22s, including physical location, length, strand, molecular weight (Mw), the number of amino acids, charge, grand average of hydropathy (GRAVY), and isoelectric point (pI), were obtained in CottonFGD through the feature analysis function (Zhu et al., 2017). Finally, the chromosome localization of *HVA22*s was mapped by TBtools and genome annotation gff3 files (Chen et al., 2020a).

### Phylogenetic analysis of HVA22s

The phylogenetic tree of *G. hirsutum*, *G. barbadense*, *G. raimondii*, *G. arboreum*, and *A. thaliana* HVA22s was built with Mega X (Kumar et al., 2018). The ClustalW with default parameters was used to align the protein sequences, and then Poisson model was employed to construct a maximum likelihood tree, and 1000 replicates bootstrap were performed. Finally, ITOL (https://itol.embl.de/) was applied to colorize this tree. All *HVA22s* were named uniformly based on evolutionary clustering results and physical location on chromosomes.

### Duplication and synteny analysis of *HVA22s*

A one-to-one comparison of the HVA22s protein sequences of *G. hirsutum*, *G. barbadense*, *G. raimondii*, and *G. arboreum* was performed by BLAST. Next, the MCScanX (http://chibba.pgml.uga.edu/duplication/) was used to calculate the four cotton species of the synteny examination of paralogous genes.

### Gene structure analysis and identification of conserved motifs

The coding sequence and full gene length of the *HVA22s* were submitted to the Gene Structure Display Server (GSDS 2.0, http://gsds.gao-lab.org/) for gene structure analysis. Multiple Em for Motif Elicitation (MEME v5.1.0, http://meme-suite.org/tools/meme) was carried out to identify the conserved protein motifs of HVA22s. The parameter was set as follow: znr (the occurrences of each functional domain in each sequence was variable) as the distribution type of the structure domain in the sequence, the width of motifs was 6–50, and 15 motifs were calculated (Bailey et al., 2009). Finally, the final data visualization was plotted by TBtools.

### Cis-acting regulatory elements analysis

A 2000 bp sequence of each *HVA22* gene upstream of the start codon was extracted from the CottonFGD database and predict the cis-acting elements contained in these sequences using the PlantCARE website (http://bioinformatics.psb.ugent.be/webtools/plantcare/html/) (Lescot et al., 2002); in addition, the visualization of cis-acting elements associated with stress response and hormonal regulation by TBtools.

### Expression profiling of *GhHVA22s*

All expression data of *HVA22s* under different stresses (blank, drought, salt, high temperature, and low temperature) were acquired from the gene expression database of TM-1(Hu et al., 2019). The expression profiles of *HVA22s* from different organs (leaf, stem, root, torus, petal, sepal, calycle, upper stamens, lower stamens, 0–5dpa unseparated ovules, and fibers, 10–25 dpa fiber, 10–20 dpa ovule) were also obtained. The expression of *HVA22s* was normalized to log_2_(FPKM), and heat maps were created with TBtools for visualization.

A 4-week-old salt-tolerant upland cotton variety Han682 was watered with 250 mM NaCl and water and was used as control group. The whole growing process of cotton keeps 16 h light/8 h dark, 25°C. At 0, 3, 6, 12, and 24 h after treatment, root tissue samples were taken for RNA extraction using the OminiPlant RNA Kit (DNase I) (CWBIO), then reverse transcribed into cDNA for qRT-PCR verification of *GhHVA22s*. Each sample was taken from two cotton seedlings, and three replicates of samples were taken at each point. The NCBI primer design website (https://www.ncbi.nlm.nih.gov/tools/primer-blast/) was used to design primers for *GhHVA22s. GhUBQ14* was used as internal control to standardize the expression of target genes. Gene expression was computed by 2^-ΔΔCt^, and Ct was the cycling threshold. The primers are listed in **Table S7**.

### Expression characterization of *GhHVA22E1D*

*GhHVA22E1D* expression characterization was performed using 4-week-old Han 682 cotton. Blank control plants were watered with water until the soil was saturated. During salt stress treatment, NaCl (250mM) solution was watered until soil saturation. For drought treatment, the soil was saturated with PEG6000 (10%) solution. The leaves were sprayed with 500 μM ABA solution for exogenous ABA treatment. Root, stem, and leaf samples were collected at 0, 3, 6, 12, and 24 h after treatment, respectively. RNA extraction, reverse transcription, and qPCR analysis were then performed, as above. The primers are listed in **Table S7**.

### Construction of plant expression vector

The *GhHVA22E1D* coding region was amplified from Han 682 using KOD polymerase (Toyobo, Shanghai, China). After double digestion with KpnI and SacI, the vector was connected to the plant overexpression vector pCAMbia2300, and 35S:: GhHVA22E1D vector was obtained (**Figure S3**).

The conserved sequence of *GhHVA22E1D* was amplified from Hand682 using KOD polymerase. The CLCrV-GhHVA22E1D vector was obtained by double digestion with SpeI and AscI and ligation to the VIGS expression vector CLCrV-00 (Gu et al., 2014). The amplification primers used are listed in **Table S7**.

### Evaluation of stress tolerance in *Arabidopsis thaliana* overexpressing *GhHVA22E1D*

*A. thaliana* plants overexpressing *GhHVA22E1D* were obtained by infecting Col-0 with flower dip method (Clough and Bent, 1998). Positive materials were obtained after kanamycin screening and PCR identification. The transformed event OE22 with the highest *GhHVA22E1D* expression was selected for purification screening, and pure T_3_ generation seeds were obtained for subsequent experiments. A light cycle of 16 h light/8 h dark was maintained throughout the reproductive period, and the temperature was always kept at 23°C.

To evaluate salt tolerance in overexpressing *GhHVA22E1D A. thaliana*, NaCl solution (200 mM) was used to water 30-day-old *A. thaliana* daily, and the relative electrical conductivity and the total chlorophyll content of rosette leaves were measured after 7 days. For drought treatment, 70 seeds each of WT and OE *A. thaliana* were sown on both sides in the same pot. After 3 weeks of normal culture, the soil was saturated with water and then naturally dried for 7 days. Then re-watering was performed, and the survival rate was counted 2 days after re-watering.

### VIGS of *GhHVA22E1D* in *G. hirsutum* and evaluation of salt tolerance and drought resistance

Cotyledons of 7-day-old cotton (Han 682) were infected with *A. tumefaciens* LBA4404 carrying the pCLCrV-*GhHVA22E1D* plasmid. The plants injected with pCLCrV-CLA1 were taken as positive control, and plants injected with pCLCrV-00 were taken as negative control. When positive control plants exhibited an albino phenotype, qPCR was performed on leaf samples injected with the pCLCrV-*GhHVA22E1D* vector to detect the silencing effect. Next, the silenced plants and negative control plants were treated with salt stress and drought stress, respectively. For salt resistance evaluation, plants were soaked with 400 mM NaCl for 10 days, then the activities of peroxidase (POD) and superoxide dismutase (SOD) and the content of malondialdehyde (MDA) in roots and leaves were determined, respectively. During the drought treatment, the watering was stopped for 2 weeks after the soil absorbed water to saturation. Next, re-watering treatment was performed and after 2 days of re-watering the survival rate was calculated.

### Determination of SOD and POD activity and MDA content

SOD activity in plant tissues was measured by photoreduction with the nitroblue tetrazolium method, the activity of POD by the guaiacol colorimetric method, and the MDA content determined by colorimetric method (Ullah et al., 2018). During the measurements, technical replicates were performed three times for each plant sample.

### Data statistics and analysis

For the sake of experimental reliability, all biological replicates and sample tests in this study were repeated three times. Finally, three replicate means and standard deviations were given.

## Results

### Genome-wide identification and characterization of the *HVA22* family in four cotton species

A total of 16 *GaHVA22s*, 17 *GrHVA22s*, 32 *GhHVA22s*, and 34 *GbHVA22s* from four cotton species were identified. The gene ID and physicochemical characteristics of all *HVA22* genes are shown in **Table S1**. The length of HVA22 proteins ranged from 140 to 418 amino acids (aa) in diploid cotton, 140 to 519 aa in *G. barabadense*, and 140 to 562 aa in *G. hirsutum*. The molecular weight ranged from 16.381 to 47.83 kDa, 16.401 to 47.779 kDa, 16.381 to 64.649 kDa, and 16.381 to 59.603 kDa, and the theoretical isoelectric point (pI) between 5.692 and 9.915, 6.262 and 9.915, 5.029 and 9.915, and 6.512 and 9.75 in GaHVA22s, GrHVA22s, GhHVA22s, and GbHVA22s, respectively.

Among all four species of cotton, the amount of *HVA22s* in the A- and D-genomes was equal or differed by one. The number of *HVA22s* in the A-genome of *G. arboreum*, *G. barbadense*, and *G. hirsutum* was 16, 17, and16, respectively; and the number of *HVA22s* in the D-genome of *G. raimondii*, *G. barbadense* and *G. hirsutum* was 17, 17, and 16, respectively. In the A-genomes of *G. barbadense* and *G. hirsutum*, there were 1, 3, 2, 1, 2, 1, 2, 1, 2, and 1 *HVA22* genes located on chromosomes A01–A13, respectively, except A02, A08, and A11. Among them, there was no *HVA22* gene on chromosomes A02, A08, and GhA11, and there was one gene on chromosome GbA11. In the D-genomes of *G. barbadense* and *G. hirsutum*, except D04 and D08, there were 1, 2, 1, 1, 2, 1, 2, 1, 1, 2 and 1 *HVA22* genes located on chromosomes D01–D13, respectively. Among them, GhD04 has one gene, GbD04 has two genes, and there was no *HVA22* gene on D08. In *G. arboreum*, there were 1, 1, 2, 2, 1, 2, 1, 1, 1, 1, 1, 1, 2, and 1 *GaHVA22s* distributed on 12 chromosomes except Chr8, respectively. There were two *HVA22* genes on each of the four chromosomes and one *HVA22* gene on each of the seven chromosomes in *G. raimondii*. The *HVA22* gene in *G. raimondii* was absent on Chr4, but had two genes on Chr8, which was opposite to the distribution of the previous three cotton species. The distribution of *HVA22s* on chromosomes is shown in **Supplementary Figure S1**.

### Phylogenetic analysis and subcellular localization prediction of *HVA22* genes in four cotton species

**Figure 1:**
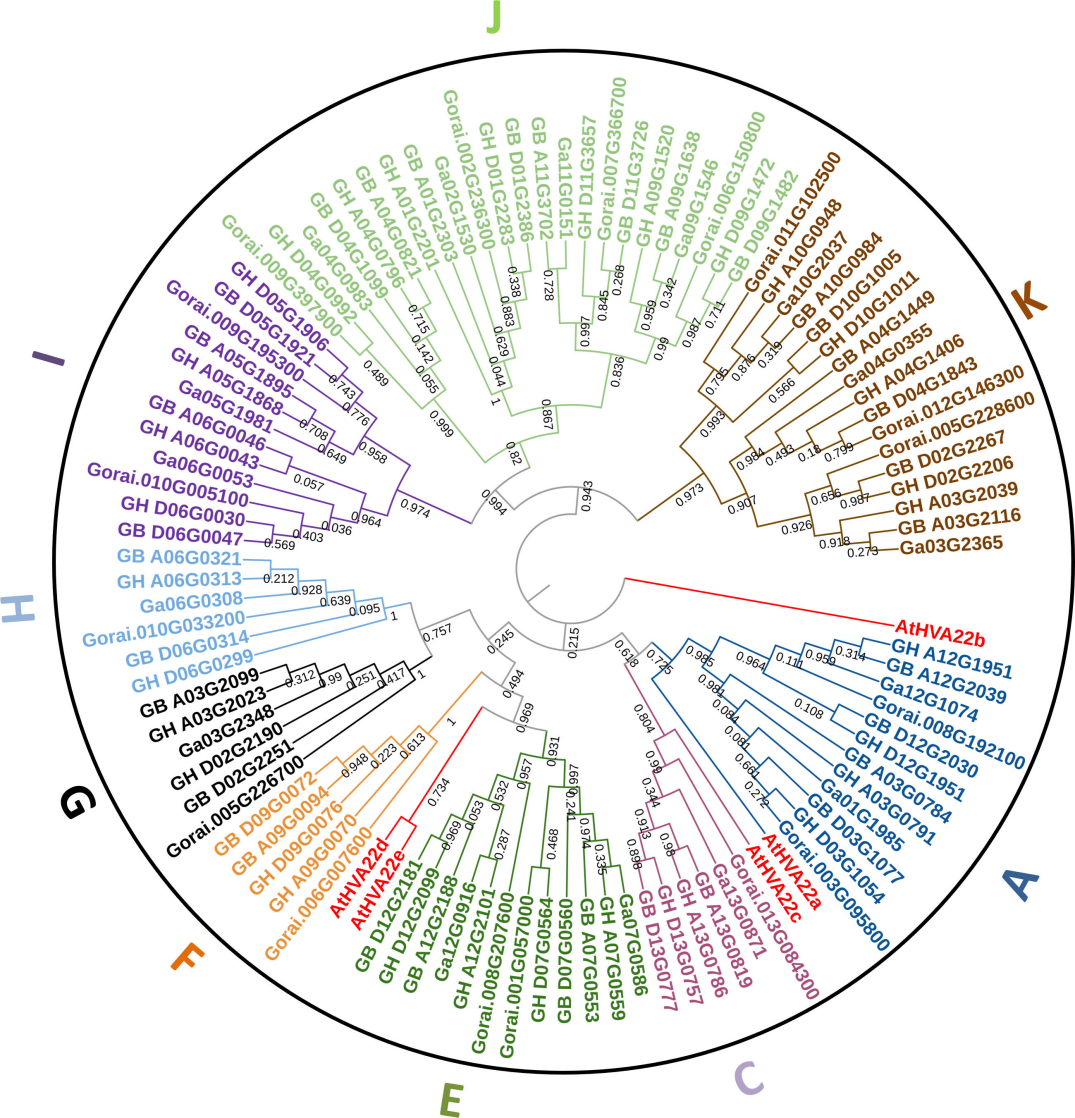
The phylogenetic tree of *HVA22s* from *A. thaliana* and four cotton species. The bootstrap value on the branch lines were from 0.03 to 1.

In the phylogenetic tree constructed based on the combination of all HVA22 protein sequences of *Arabidopsis thaliana* and four cotton species, all cotton HVA22s were classified into nine lineages (A, C, E, F, G, H, I, J, and K) without B and D lineages (**Figure 1**).The number of *HVA22* genes varied greatly among different lineages, with the J lineage containing the most genes (23 genes) and the F lineage containing the fewest genes (five genes). In the terminal branch of the phylogenetic tree, three genes from the A-genome or D-genome were clustered together, with the exception of one different branch in each of the F, G, and K lineages. The three genes in each group were from two tetraploid species and one diploid species. This is consistent with the previous finding that the A-genome and D-genome of tetraploid cotton were formed by hybridization and doubling of two diploid cotton species (Wendel and Cronn, 2003; Huang et al., 2020). All *HVA22* genes were named uniformly according to their physical location and lineage classification (**Table S1**).

The predicted results of subcellular localization of HVA22 protein are listed in **Table S1**. Most cotton HVA22s were predicted to be localized to the plasma membrane, while a minority was localized to the cytoplasmic, extracellular space, chloroplast, nuclear, and mitochondria. The proteins in the A, C, E, F, and H lineages were all localized on the plasma membrane, while the proteins of the G lineage proteins were all localized on extracellular space, while the I, J, and K lineages had 8, 5, and 3 proteins with multiple localization sites, respectively. The differences of subcellular localization implied that there might be a diversity of functions of HVA22s.

### Synteny and duplication analysis of *HVA22*s

**Figure 2:**
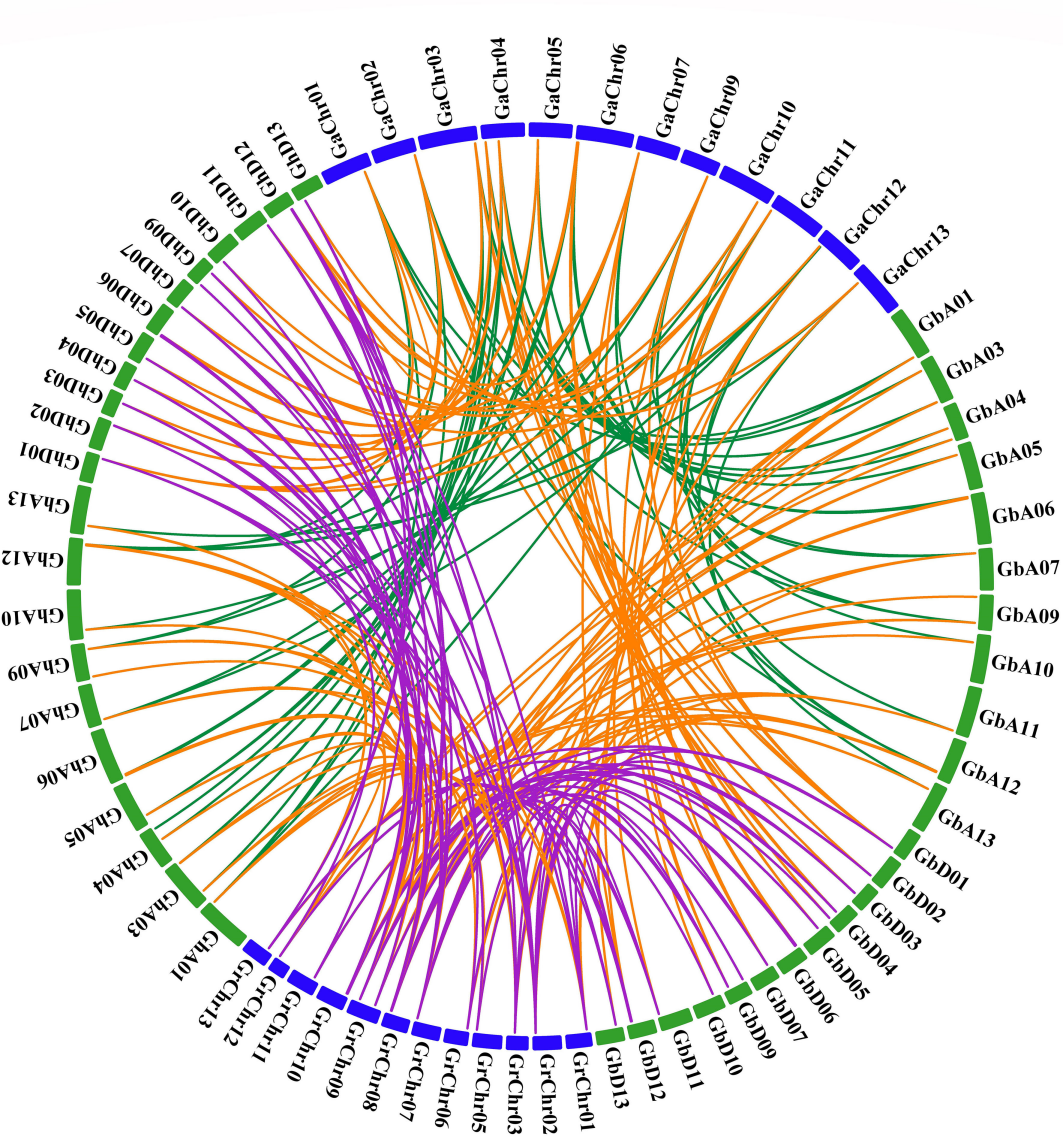
The synteny and duplication of *HVA22s* among four cotton species.

In order to show the synteny relationship, all *HVA22s* of the four cotton species were compared and aligned for homology, and a synteny relationship plot was constructed (**Figure 2**). There were 30, 32, and 32 genes homologous to 32 genes of *G. hirsutum* in *G. barbadense*, *G. raimondii*, and *G. arboreum*, respectively, and there were 30 *GhHVA22s* that were common homologous in all three cotton varieties (**Table S2**).

During the evolution of cotton, the included proximal, duplicate mechanisms, dispersed and tandem duplications, and segmental duplications or whole-genome duplications (WGD) played a crucial role in the expanded membership of gene families (Chen et al., 2019; Wang et al., 2019). The duplications of *HVA22s* were classified as segmental repeats or WGD in both tetraploid cotton (*G. hirsutum* and *G. barbadense*) (**Table S3**), but in diploids (*G. raimondii* and *G. arboreum)*, three *GaHVA22s* (Ga01G1985, Ga04G0983, and Ga10G2037) and two *GrHVA22s* (Gorai.006G007600 and Gorai.011G102500) were identified as dispersed duplications, and the rest were segmental duplications or WGD. This suggests that the expansion and evolution of the *HVA22* gene family in cotton may be primarily driven by segmental duplications or WGD.

In order to understand the co-linearity of all *HVA22* genes among the four cotton species, all linked gene pairs were identified (**Figure 2**). Corresponding to *G. arboreum*, 26 and 25 co-linear gene pairs were characterized in the A-genome of two tetraploids (*G. barbadense* and *G. hirsutum*), respectively, and 27 co-linear gene pairs were characterized in the d-genome of both tetraploids. Furthermore, in contrast to *G. raimondii*, there were 36 and 31 co-linear gene pairs in the two tetraploid A-genomes and 39 and 37 co-linear gene pairs in the D-genome, respectively. Moreover, 33 collinear gene pairs were co-linear between the two diploids and 109 pairs were identified between the two tetraploids.

### Structure and conserved motif of cotton *HVA22s*

**Figure 3:**
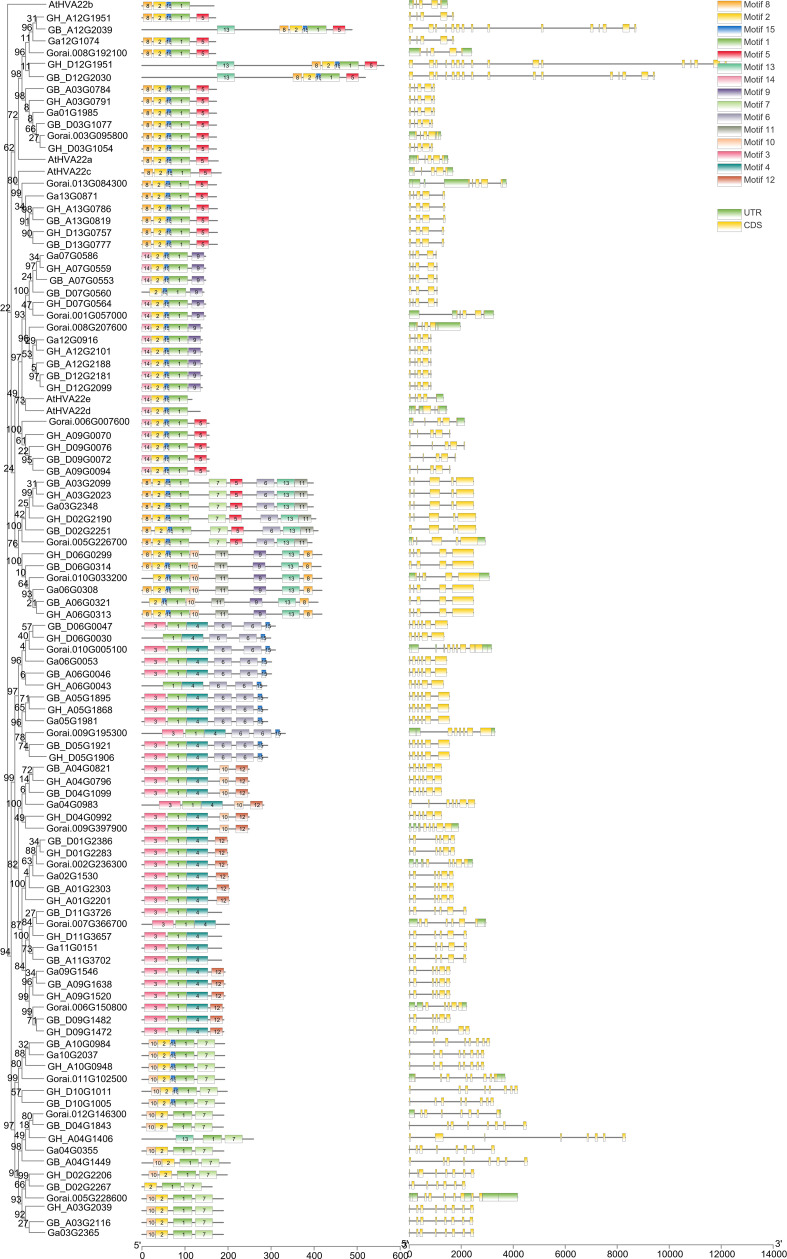
Gene structure and conserved motif of HVA22s in G. hirsutum, G. barbadense, G. arboreum, G. raimondii, and A. thaliana.

The structural map of the *HVA22s* indicated the distribution of the coding regions (**Figure 3**). Introns were present in the *HVA22s* of all four cotton species. There were 10.1% of *HVA22s* with four exons, which were scattered in C, E, and G lineages. Five exons were found in 36.4% *HVA22s*, mostly concentrated in A, C, F, E, and H lineages. There were 32.3% of *HVA22s* that had six exons. These were concentrated in I and J lineages. The remaining 21.2% of the genes had six or more exons. All genes of the K lineages had this characteristic. In addition, untranslated regions (UTRs) were present in all 17 *HVA22* genes of *G. raimondii*. The gff3 files provided by the genomic databases used in this report for *G. hirsutum*, *G. barbadense*, and *G. arboreum* had no UTR annotations, so the distribution of their UTRs was not shown here.

Conserved motif analysis of the full-length sequence of the HVA22 protein revealed that 15 amino acid conserved motifs were identified in all HVA22s (**Table S4** and **Figure 3**).The conserved motifs of cotton HVA22 totaled three to nine, and motif 1 was a conserved motif shared by all HVA22 proteins. Furthermore, motif 2 and motif 15 were conserved in the A, C, E, F, G, and H lineages, and motif 12 was specific to the J lineages. The number and type of conserved motifs of genes were similar in the same lineage. This supported the reliability of the evolutionary tree that was constructed in this paper.

### Analysis of the cis-acting element of the cotton *HVA22s*

**Figure 4:**
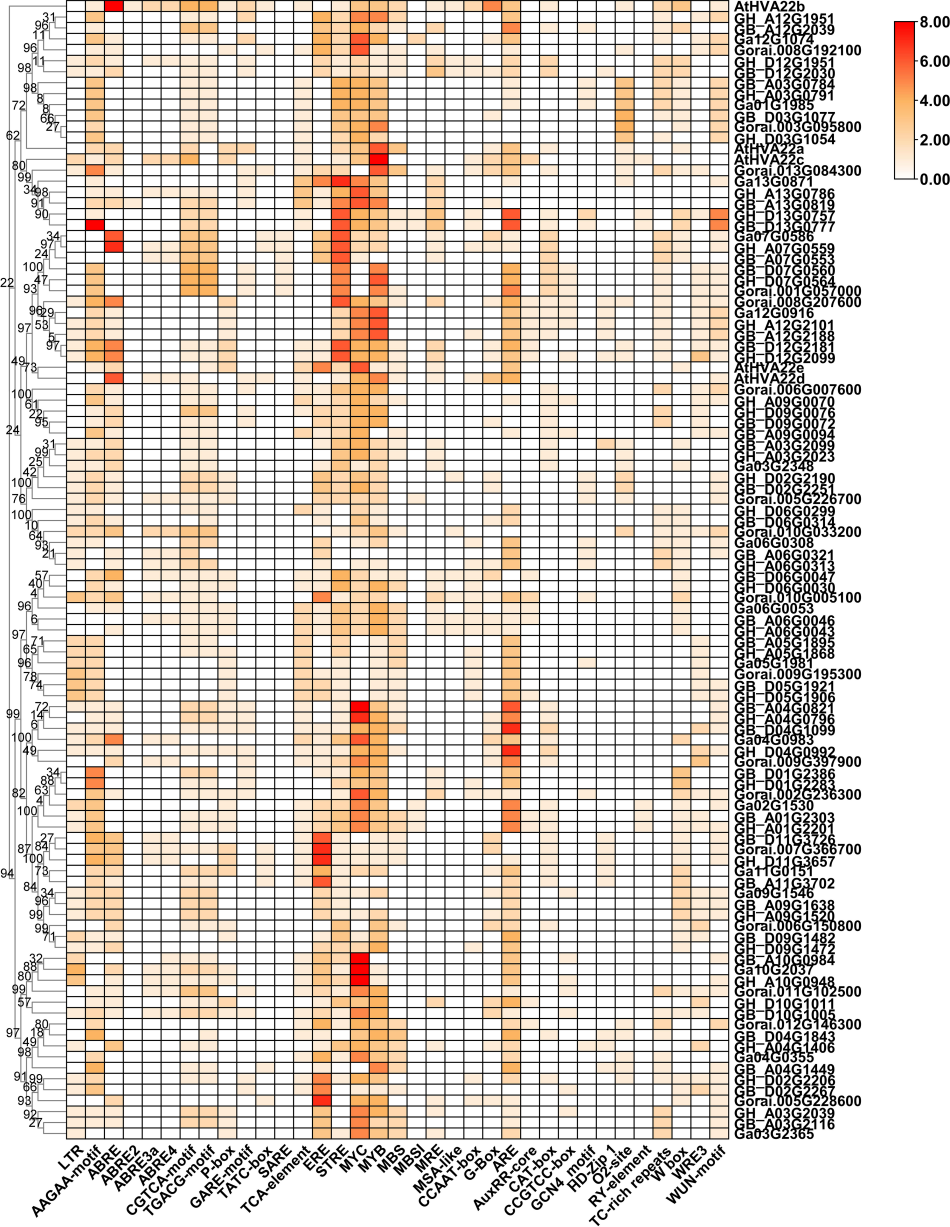
Predicted hormone response, stress response, and growth-related cis-elements in the promoter region of *HVA22* genes.

The promoter prediction results showed that numerous drought-responsive elements (MYB) and light-responsive elements (BOX 4, Sp1, I-box, and G-box) were found in all *HVA22*s promoter regions (**Table S5** and **Figure 4**). In addition, heat maps were used to visualize cis-acting elements associated with hormone regulation and stress response. There were a variety of phytohormone responsive elements in the *HVA22* genes promoter, including the ABA response element (ABRE), ethylene response element (ERE), Methyl Jasmonate response element (CGGTA-motif and TGACG-motif), gibberellin response element (GARE-motif, P-box and TATC-box), and salicylic acid-responsive elements (SARE and TCA-element), which showed that the *HVA22s* expression was subject to the regulation of different phytohormones. Some stress-responsive elements were also found, such as low temperature response element (LTR), defense and stress response elements (TC-rich repeats), anaerobic inducible element (ARE), and wound response elements (WUN and WRE3 motifs). In addition, several growth and development elements were predicted, including meristem expression elements (CCGTCC-motif and CAT-box) and endosperm expression elements (GCN4-motif). This result showed that *HVA22s* were involved in the growth of cotton and its response to environmental stresses. Different lineages had disparate gene promoter characteristics. For example, the promoters of the C and E lineages generally contained a large number of osmotic stress response elements (STRE), while the promoters of J and K lineages had a great deal of ethylene response elements (ERs). This suggested that the expression of *HVA22s* in different subgroups was subject to disparate regulatory factors.

### Expression profiling of *GhHVA22*s under various stress and in different tissues

**Figure 5:**
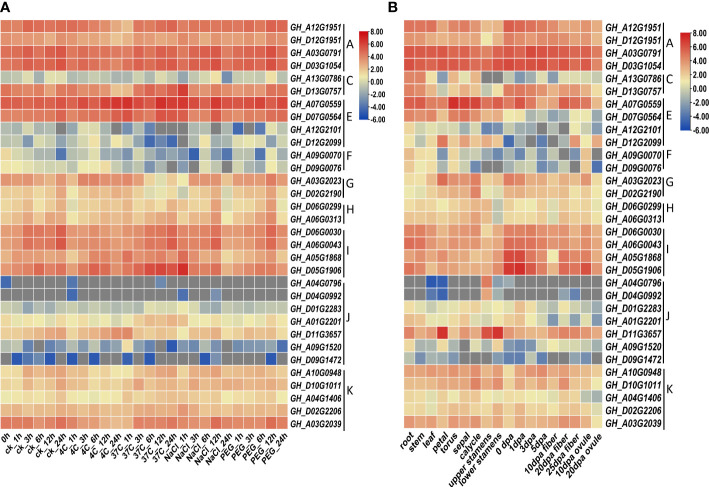
Expression pattern analysis of *GhHVA22*. **(A)** Expression pattern analysis of *GhHVA22* under control, low temperature, high temperature, salt and drought stress conditions, expressed as CK, 4°C, 37°C, 400mM NaCl and 20% PEG, respectively. **(B)** Analysis of the expression pattern of *GhHVA22* in different organs, where 0-5 dpa represents ovules and fibers that were not isolated for 1–5 days.

Heat map of gene expression shows that the expression patterns of homologous genes were relatively similar under stress conditions, except for the two homologous genes of the C lineage (**Figure 5**; **Table S6**). Genes in the A lineage were all highly expressed, while most genes in the F and J lineages were less expressed. The response patterns of different *HVA22s* genes to environmental stress were not necessarily the same. For example, the *GhHVA22F1A* (*GH_A09G0070*) gene in the F lineage showed a down-regulated expression pattern in response to stress, while the *GhHVA22E1D* (*GH_D07G0564*) gene in the E lineage showed an up-regulated expression pattern under stress conditions. The expression of the same gene was not the same in different plant tissues. For instance, the *GhHVA22E1D* gene was more highly expressed in tissues such as rhizomes, leaves, and flowers, and less highly expressed in ovules and fibers.

**Figure 6:**
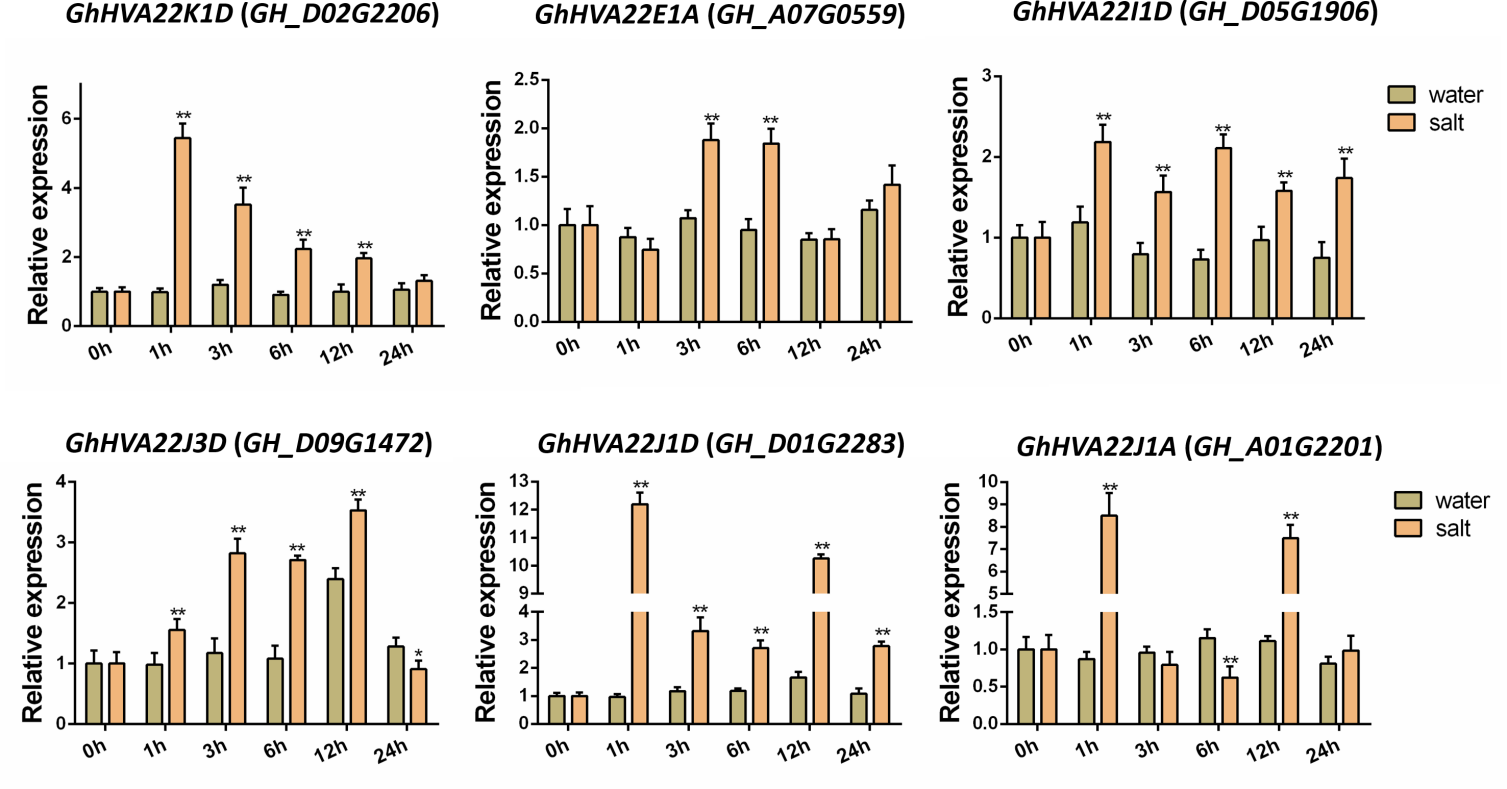
Expression patterns of six *GhHVA22* genes in response to salt treatment. Each sample was sampled at 0, 3, 6, 12 and 24h after treatment. The results are the mean of three replicates. Significant differences are indicated by * (P < 0.05) and ** (P < 0.01, Student T-test).

To further validate the ability of *GhHVA22s* to respond to salt stress, six *GhHVA22s* in the TM-1 database that responded to salt stress were randomly selected for qRT-PCR validation. All six genes were able to rapidly up-regulate their expression after salt stress (**Figure 6**). Among them, *GhHVA22K1D* (*GH_D02G2206*), *GhHVA22I1D* (*GH_D05G1906*), *GhHVA22J1D* (*GH_D01G2283*), and *GhHVA22J1A* (*GH_A01G2201*) had the highest expression at 1 h after salt treatment (250 mM NaCl). The expression level of *GhHVA22K1D* increased to the highest level at 1 h after salt stress and then gradually decreased until the expression level was similar to the control. The expression of *GhHVA22J3D* (*GH_D09G1472*) was gradually up-regulated after salt stress, and the expression level was the highest at 12h. *GhHVA22I1D* and *GhHVA22J1D* maintained high levels of expression within 24 h after salt stress. In contrast, *GhHVA22J1A* was up-regulated only at 1 h and 12 h and showed a down-regulated expression pattern at 6 h after salt treatment. *GhHVA22E1A* (*GH_A07G0559*) was up-regulated only at 3 h and 6 h after salt treatment. From these results, most of the genes in the *HVA22* family were able to respond to salt stress through expression regulation.

### Expression pattern of *GhHVA22E1D*

**Figure 7:**
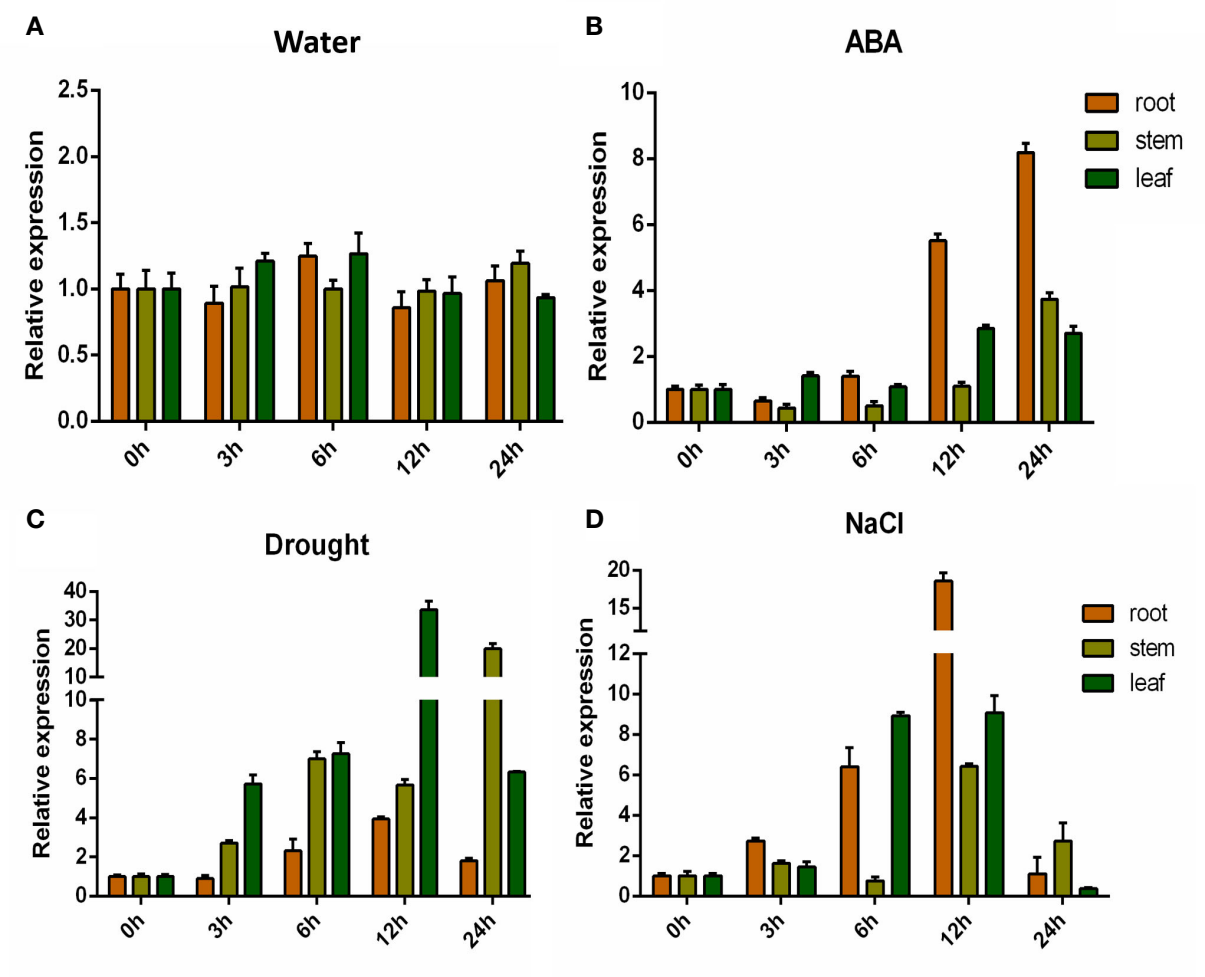
Expression pattern *of GhHVA22E1D*. Expression pattern of *GhHVA22E1D* in *G hirsutum* after 500 μM ABA **(B)**, 10% PEG6000 **(C)** and 250 mM NaCl **(D)** stresses, and the water treatment group was the control **(A)**. Each sample was sampled at 0, 3, 6, 12, and 24 h after treatment. The results are the mean of three replicates.

Previously, whole-genome association and differential expression analysis of salt tolerance at germination stage in upland cotton were performed in our laboratory. The expression of the *GhHVA22E1D* (*GH_D07G0564*) gene, which is related to salt tolerance, was detected to be up-regulated under salt stress (Yuan et al., 2019). Therefore, we performed a more detailed expression pattern detection for *GhHVA22E1D*. *GhHVA22E1D* was up-regulated in leaves, stems, and roots under exogenous ABA, salt stress, and drought stress (**Figure 7**). *GhHVA22E1D* showed the highest sensitivity to exogenous ABA and salt stress in root tissue. The expression of *GhHVA22E1D* was up-regulated to 8.2-fold of the control at 24 h after ABA treatment, and the expression level of *GhHVA22E1D* was up-regulated to 18.6-fold in the roots after salt treatment for 12 h. *GhHVA22E1D* in leaf tissue and stem tissue was more sensitive to drought stress, reaching 33.7-fold and 19.9-fold that of the control at 12 h and 24 h after drought stress, respectively.

### Ectopic overexpression of *GhHVA22E1D* enhances tolerance of *Arabidopsis thaliana* to abiotic stresses

**Figure 8:**
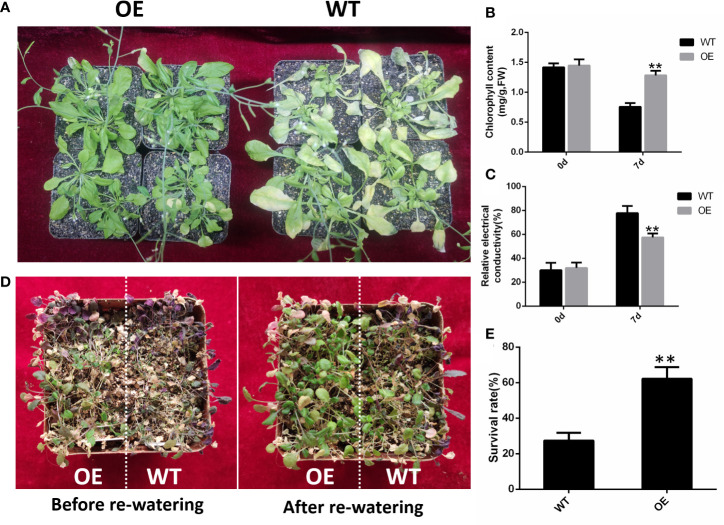
The overexpression of *GhHVA22E1D* increased salt tolerance and drought resistance of *Arabidopsis thaliana*. **(A)** Phenotype of Arabidopsis plants after 7 days of watering with 200 mM NaCl solution. **(B)** Total chlorophyll content in Arabidopsis leaves after 0 and 7 days of salt stress. **(C)** Relative electrical conductivity of Arabidopsis leaves after 0 and 7 days of salt stress. **(D)** Phenotype of Arabidopsis after 7 days of natural drought and 2 days of re-watering. **(E)** Survival rate of Arabidopsis after 2 days of re-watering. Results are from three replicate experiments. Significant differences are indicated by **(p < 0.01, Student *t*-test).

*GhHVA22E1D* was overexpressed in *A. thaliana* and a homozygous T_3_ generation was obtained. The OE22 line with the highest gene expression was selected to identify the salt and drought tolerance of the plant (**Figure S2**). After 7 days of irrigation with 200 mM NaCl solution, the leaves of OE plants died less, while the rosette leaves of WT plants had yellowed and died in large areas (**Figure 8A**). Chlorophyll content in the leaves of OE plants was significantly higher than that of WT plants after salt stress treatment (**Figure 8B**), and conductivity was significantly lower than that of control plants (**Figure 8C**). After watering was stopped for 7 days, almost all the leaves of the WT plants were wilted, while OE plants still had some healthy leaves (**Figure 8C**). After re-watering, the survival rate of OE plants was 62.26%, which was significantly higher than that of wild-type plants (27.42%) (**Figure 8D**). These results indicated that the overexpression of *GhHVA22E1D* could significantly improve the salinity and drought resistance in *A. thaliana* plants.

### Silencing *GhHVA22E1D* by VIGS reduced salt and drought tolerance in *G. hirsutum*

**Figure 9:**
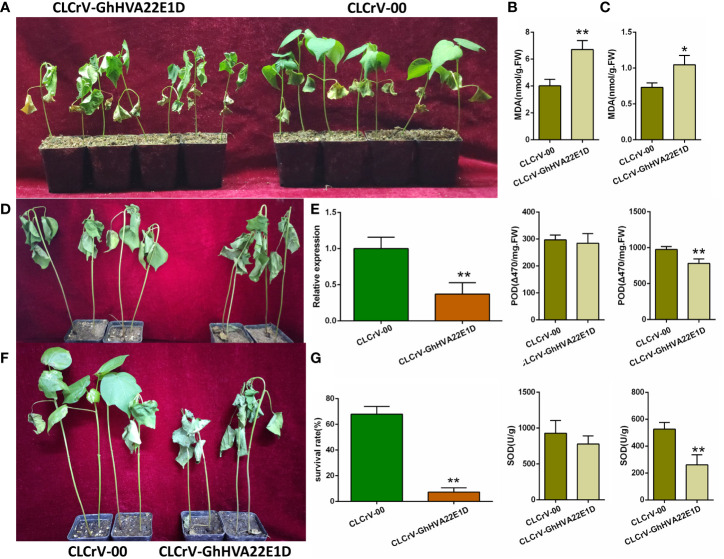
Silencing of *GhHVA22E1D* leads to reduced salt and drought tolerance in *G hirsutum*. **(A)** Phenotype of cotton after soaked in 400 mM NaCl solution for 10 days. Detection of POD and SOD activities and MDA content in cotton leaves **(B)** and roots **(C)** after salt stress. **(D)** The phenotype of cotton after 14 days of natural drought. **(E)** Silencing efficiency of CLCrV- GhHVA22E1D plants. Phenotype **(F)** and survival rate **(G)** of cotton plants after 2 days of re-watering. The results are the mean of three replicates. *p ≤  0.05, **p ≤ 0.01 (Student *t*-test).

CLCrV-based virus-induced gene silencing (VIGS) was used to silence the *GhHVA22E1D* gene in *G. hirsutum*. After 2 weeks of *A. tumefaciens* injection, the *GhHVA22E1D* transcript level of CLCrV-GhHVA22E1D plants decreased by 62.79%, indicating that this gene was effectively silenced (**Figure 9E**). CLCrV-GhHVA22E1D plants wilted most of the whole plant after 10 days of 400 mM NaCl stress, while only a few leaves of the control plants wilted (**Figure 9A**). The activities of POD and SOD in root tissues of CLCrV-GhHVA22E1D plants were significantly lower, and the MDA content was significantly higher than that of the control under salt stress (**Figure 9C**). In the leaves, the MDA content of the silenced plants was significantly higher than that of the control after salt treatment, while the activities of POD and SOD were similar to those of the control plants (**Figure 9B**). It suggests that the role of *GhHVA22E1D* in root and leaf tissues was not exactly the same when plants were subjected to salt stress. Both plants were severely wilted after 14 days of natural drought (**Figure 9D**). But after 2 days of re-watering, most of the empty vector control plants were able to recover, while most of the CLCrV-GhHVA22E1D plants wilted and died (**Figure 9F**). From the statistics of the survival rate, it could be seen that the survival rate of the silenced group was 7.31%, which was significantly lower than that of the control group, which was 67.97% (**Figure 9G**). The above results showed that the silencing of *GhHVA22E1D* significantly reduced the salt tolerance and drought resistance of cotton.

## Discussion

Here, 16 *GaHVA22s*, 17 *GrHVA22s*, 32 *GhHVA22s*, and 34 *GbHVA22s* genes were identified. The *HVA22* genes in cotton were clustered into nine lineages, which were different from five lineages in *A. thaliana* (Chen et al., 2002), 15 lineages in tomato (Wai et al., 2022) and six lineages in citrus (Gomes Ferreira et al., 2019), indicating that *HVA22* classes were different among different species. In each lineage, the number of *HVA22* genes in tetraploid cotton was almost twice that of diploid cotton. And in the same chromosome number, the tetraploid *HVA22* and the diploid *HVA22* clustered together. A_0_ and D_5_ genomes hybridized, doubled, and differentiated to form the AD_1_ and AD_2_ genomes of *G. hirsutum* and *G. barbadense*, and A_0_ differentiated to the A_2_ genome of *G. arboreum* (Huang et al., 2020). The genome of the four cotton species was traced back to the common A_0_ and D_5_ ancestor, and the distribution pattern of HVA22 family genes also confirmed this conclusion.

The similarity in gene structure and distribution of conserved motifs in the same subclade suggested that they had similar roles in plant growth (Zafar et al., 2022). The distribution of motifs varied among different subclades. Some motifs were even specific to a particular subclade; for example, motif 12 was specific to J lineage. This suggested that gene functions differed between subgroups (Sun et al., 2020). Cis-acting elements on gene promoters play an active role in the stress response of plants, and the type of cis-acting element determines the signals to which a gene can respond (Zhao et al., 2016). Multiple cis-acting elements that respond to environmental stress (ARE, STRE, MBS, LTR, and WRE3, etc.), phytohormones (ABRE, AAGAA-motif, TGACG-motif, CGTCA-motif, TCA-element and P-box, etc.), and those responsible for growth and development (RY-element, HD-Zip 1, CCGTCC-box and CAT-box, etc.), respectively, were found in the promoter sequences of the *HVA22* family of cotton (Zhu et al., 2005; Faraji et al., 2018; Yang et al., 2019; Zhang et al., 2019). In addition, a large number of MYC and MYB transcription factor binding sites existed in most *HVA22* gene promoters. There was evidence that MYC and MYB transcription factors play an important role in plant resistance to stress (Wang et al., 2021; Kim et al., 2022; Wang et al., 2022). Previous studies showed that the inducible *HVA22* promoter could respond to salt, drought, low temperature, and abscisic acid induction, which supported the accuracy of our results (Chen et al., 2002; Xiao et al., 2009; Chen et al., 2014; Cheng et al., 2021; Lyu et al., 2021). The distribution of these cis-acting elements on the promoter indicates that *HVA22s* was likely to respond to adversity stress.

The study of gene expression patterns was usually used to predict gene function. The expression of *HVA22* was different in different crops. For example, the *HVA22* gene in maize was down-regulated after cold stress (Chen et al., 2014), while the *CmHVA22* gene in pumpkin (*Cucurbita moschata*) was up-regulated after cold stress (Wang, 2021). The *HVA22* gene response patterns of different subgroups in *G. hirsutum* differed in response to environmental stress. For example, the genes of the K lineage responded rapidly and showed a down-regulated expression pattern in response to cold stress, while the genes of I lineage were up-regulated under heat stress. *GhHVA22Is* tended to be expressed in ovules and fibers, which was consistent with the previous detection of differential expression of the *HVA22* during seed development in *Jatropha curcas* (Maghuly et al., 2020). On the contrary, *GhHVA22Es* were more favored in vegetative tissues, consistent with the pattern previously found in citrus (Gomes Ferreira et al., 2019). Several duplication events of HVA22 family genes observed in the cotton genome, such as *GhHVA22C1A* and *GhHVA22C1D*, showed different expression patterns. It seems that during evolution, some intentional point mutations occurred in the coding sequence and promoter region of the gene, resulting in a change in the expression pattern of duplicated genes (Heidari et al., 2022; Yaghobi and Heidari, 2023). Further qRT-PCR analysis demonstrated that *GhHVA22s* could be rapidly upregulated in response to salt stress. In our qRT-PCR results, the level of up-regulated expression of *GhHVA22s* in response to salt stress appeared to be more significant than the differences in expression profiles. This may be because the data in the expression profile came from the whole cotton seedling after salt treatment, while the root tissue after salt stress was adopted in this experiment. The expression trend of *GhHVA22s* in root in response to salt stress may be slightly different from that in other tissues. This point was confirmed by qRT-PCR analysis of root, stem, and leaf tissues of *GhHVA22E1D* in response to salt stress.

The *GhHVA22E1D* of the E lineage was previously identified as a differentially expressed gene under salt stress in the association analysis of the salt-tolerant traits and salt-tolerance transcriptome in *G. hirsutum* seedling stage (Yuan et al., 2019). In this experiment, *GhHVA22E1D* was up-regulated under salt stress and drought stress, which verified the previous findings. In addition, *GhHVA22E1D* was also up-regulated in cotton under exogenous ABA stress, which confirmed that *HVA22* gene was an ABA responsive gene (Chen et al., 2002). Arabidopsis overexpressed *GhHVA22E1D* exhibited significant salt tolerance and drought resistance, while cotton silenced *GhHVA22E1D* showed significantly reduced salt and drought tolerance. These results strongly support the conclusion that *GhHVA22E1D* is a salt tolerance responsive gene. In addition, the mechanism of *GhHVA22E1D* response to stress in different tissues of cotton seems to be different. For example, in roots, differential expression of *GhHVA22E1D* under salt stress regulated the activity of POP and SOD and affected the accumulation of MDA content, whereas in leaves, differential expression of *GhHVA22E1D* only seemed to cause differences in the accumulation of MDA content. From the perspective of gene expression pattern, *GhHVA22E1D* gene was most sensitive to salt stress in roots and drought stress in leaves. This might be related to the different functions of different organs in response to stress. Roots were first subjected to salt stress, and plants can resist salt stress by regulating the absorption of ions and water in root tissues (Hines, 2008; Rajabi et al., 2014; Deolu-Ajayi et al., 2019). When the plant was suffered from drought stress, they first reduce water loss through stomatal regulation (Tardieu et al., 2018; Visentin et al., 2020; Ghimire et al., 2021). Finally, whether *GhHVA22E1D* had a crucial role in these processes needs further verification.

## Conclusion

In the present study, there were 16 *GaHVA22s*, 17 *GrHVA22s*, 32 *GhHVA22s*, and 34 *GbHVA22s* genes identified, and these *HVA22s* were divided into nine lineages. The gene structures and conserved motifs of these *HVA22s* had significant similarity in the same lineage, and thus genes of the same lineage had similar functions. Six *GhHVA22s* could be rapidly up-regulated in response to salt stress. Overexpression of *GhHVA22E1D* in *A. thaliana* improved salt tolerance and drought resistance of Arabidopsis. Conversely, silencing *GhHVA22E1D* in cotton decreased salt tolerance and drought resistance of cotton. These results provide a basis for further understanding the role of *HVA22s* in plant resistance to high salt and drought stress. These results would be helpful in the future for genomics-assisted breeding programs for salt and drought tolerance in cotton.

## Data availability statement

The datasets presented in this study can be found in online repositories. The names of the repository/repositories and accession number(s) can be found in the article/**Supplementary Material**.

## Author contributions

XLS and ZW: Designed experiments, revised manuscripts, and obtained funds. HZ: Completed gene function identification and manuscript writing. YY and QW: Performed the bioinformatics analysis. HX and MX: Data statistical analysis. MS: Corrected the grammar of the manuscript. TZ, XZ, LM, and XZS: Plant material management. All authors contributed to the article and approved the submitted version.
