# Supplementary material for: Genome-wide identification and expression analysis of the HVA22 gene family in cotton and functional analysis of GhHVA22E1D in drought and salt tolerance
Source: Front Plant Sci. 2023 Mar 6;14:1139526. doi: 10.3389/fpls.2023.1139526 (PMC10025482; doi:10.3389/fpls.2023.1139526)
Supplement: Supplementary file 1 [file DataSheet_1.docx]

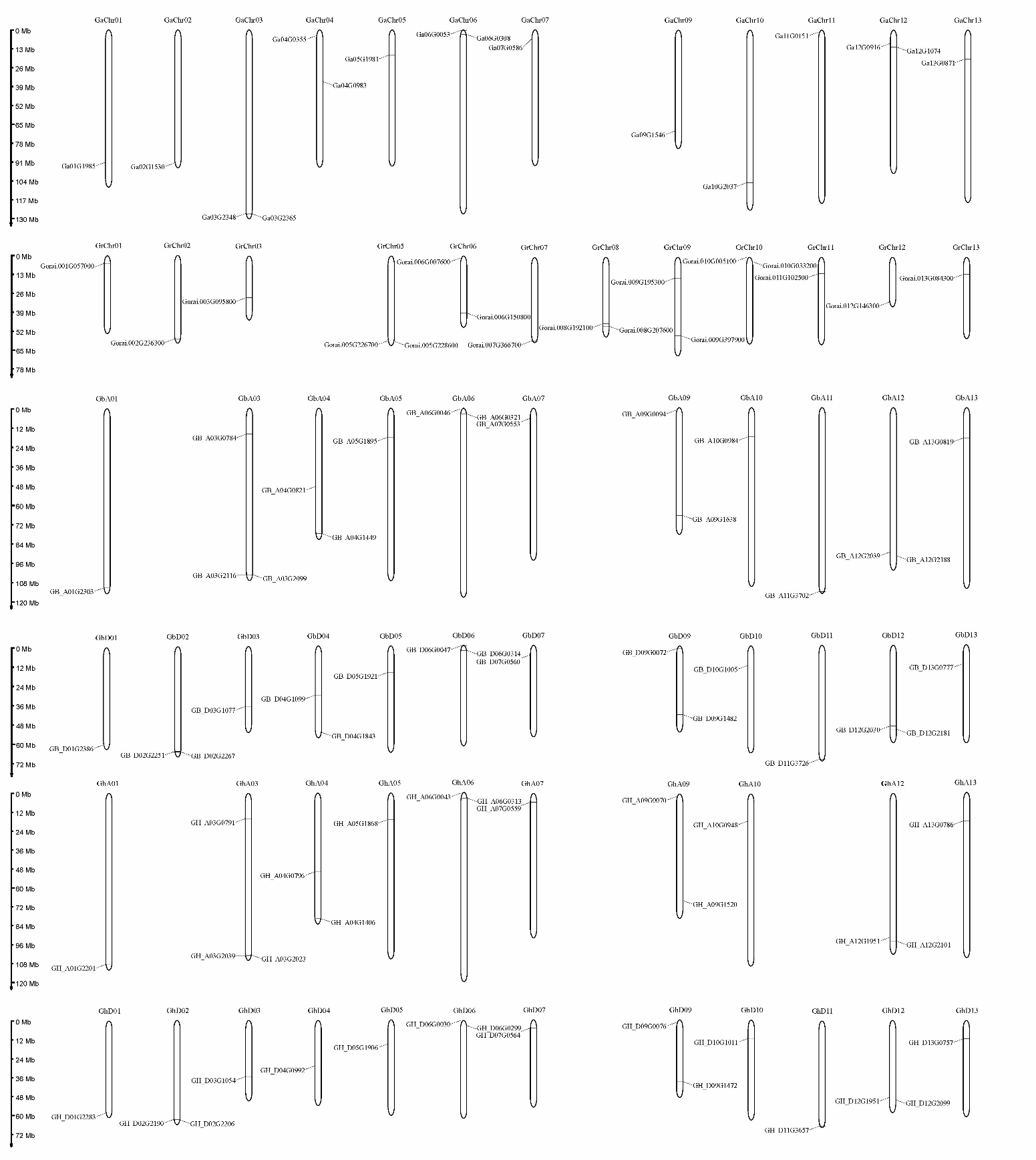


Figure S1 Distribution of HVA22s on cotton chromosomes


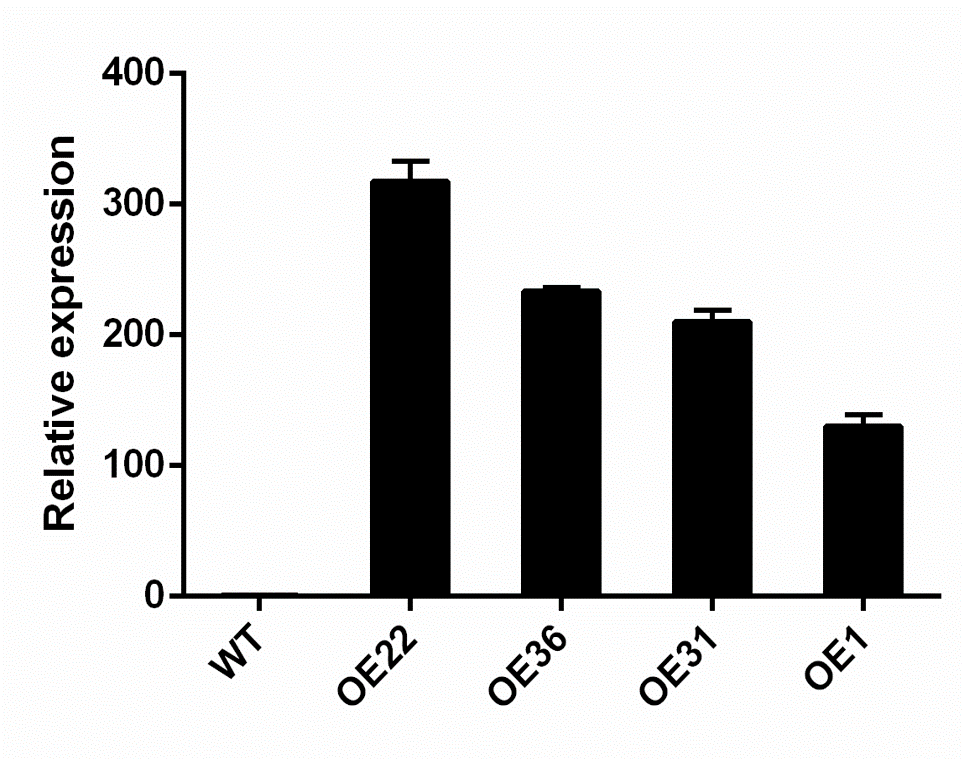


Figure S2 Identification of *GhHVA22E1D* gene expression in transgenic Arabidopsis lines. Values are means of three replicates.


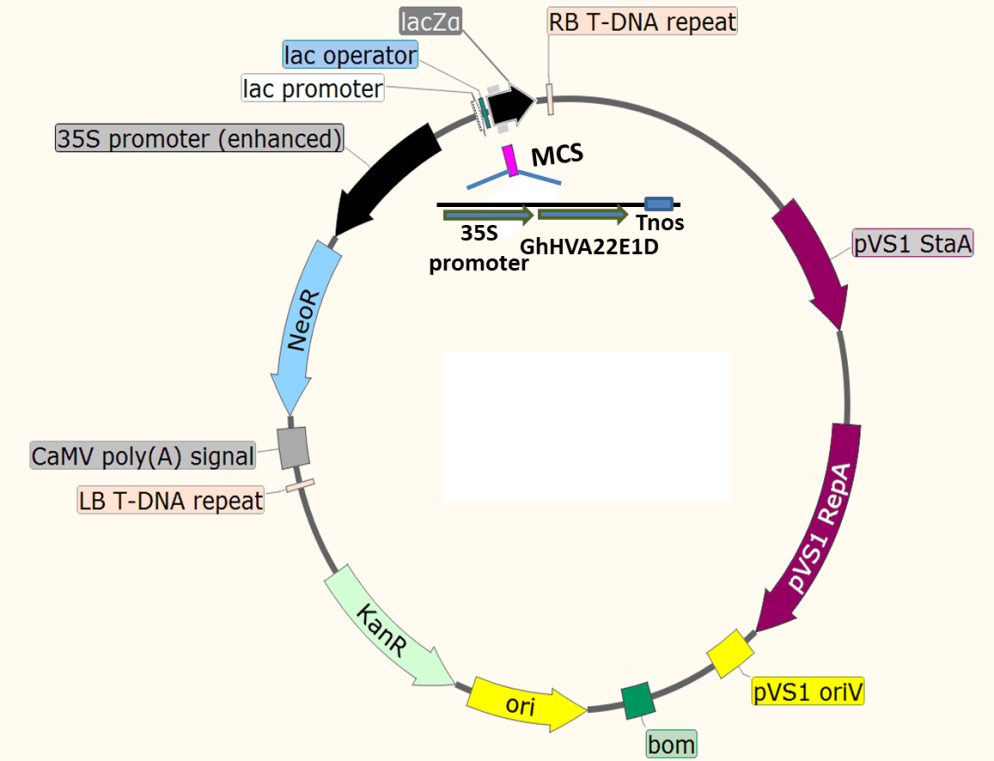


Figure S3 The map of 35S:: GhHVA22E1D vector. The sequence of the green arrow inside the circle is the inserted sequence, and the direction of the arrow represents the direction of the inserted sequence.
